# Hes-6, an inhibitor of Hes-1, is regulated by 17β-estradiol and promotes breast cancer cell proliferation

**DOI:** 10.1186/bcr2446

**Published:** 2009-11-05

**Authors:** Johan Hartman, Eric W-F Lam, Jan-Åke Gustafsson, Anders Ström

**Affiliations:** 1 Department of Biosciences and Nutrition, Karolinska Institutet, Nobels väg 5, Solna Alfred Nobels Allé 8, 141 57 Huddinge, Sweden; 2 Cancer Research-UK Labs, Department of Oncology, MRC Cyclotron Building, Imperial College London, Hammersmith Hospital Campus, Du Cane Road, London W12 0NN, UK; 3 Center for Nuclear Receptors and Cell Signaling, Department of Biology and Biochemistry, University of Houston, 4800 Calhoun Road, Houston, TX 77204, USA

## Abstract

**Introduction:**

Hes-6 is a member of the basic helix-loop-helix (bHLH) family of transcription factors, and its overexpression has been reported in metastatic cancers of different origins. Hes-6 has been described as an inhibitor of Hes-1 during neuronal development, although its function in cancer is not known. In this study, we investigated the function of Hes-6 in breast cancer and tested the hypothesis that Hes-6 enhances breast cancer cell proliferation and is regulated by estrogen.

**Methods:**

To investigate the function of Hes-6, T47D cells stably expressing Hes-6 were generated by lentiviral transduction, and conversely, siRNA also was used to knock down Hes-6 expression in breast cancer cells. The Hes-6-expressing T47D cells were transplanted into immunodeficient mice to study effects on tumor growth.

**Results:**

We found that Hes-6 expression was significantly higher in the high-grade, estrogen receptor (ER)α-negative SKBR3 and MDA-MB-231 cells compared with the ERα-positive, non-metastasizing T47D and MCF-7 breast carcinoma cells. Moreover, the level of Hes-6 mRNA was 28 times higher in breast cancer samples compared with normal breast samples. In Hes-6-expressing T47D cells, Hes-6 ectopic expression was shown to stimulate cell proliferation *in vitro *as well as breast tumor growth in xenografts. Moreover, expression of Hes-6 resulted in induction of *E2F-1*, a crucial target gene for the transcriptional repressor Hes-1. Consistently, silencing of Hes-6 by siRNA resulted in downregulation of E2F-1 expression, whereas estrogen treatment caused induction of Hes-6 and downstream targets hASH-1 and E2F-1 in MCF-7 cells.

**Conclusions:**

Together, the data suggest that Hes-6 is a potential oncogene overexpressed in breast cancer, with a tumor-promoting and proliferative function. Furthermore, *Hes-6 *is a novel estrogen-regulated gene in breast cancer cells. An understanding of the role and regulation of *Hes-6 *could provide insights into estrogen signaling and endocrine resistance in breast cancer and, hence, could be important for the development of novel anticancer drugs.

## Introduction

The majority of breast cancer cells are dependent on estrogens to support their survival and proliferation [1]. 17β-Estradiol (E_2_) is the most potent estrogen as well as the predominant estrogen in premenopausal women. In breast cancer, two main types of estrogen receptors (ERs) exist, ERα and ERβ [2-4]. As shown by *in vitro *experiments, ERα mediates the proliferative effect of estrogens, whereas ERβ inhibits proliferation [5] in breast cancer cells. In T47D and MCF-7 breast cancer cells, ERα promotes proliferation by stimulating expression of cell-cycle regulators and through downregulation of the transcriptional repressors, such as Hes-1. Hes-1 is a member of the basic helix-loop-helix (bHLH) family of transcription factors [6], first described in embryonic development, in which Hes-1 inhibits differentiation of developing neurons. In breast cancer cells, downregulation of Hes-1 is essential for estrogen-mediated proliferation [7]. Consistently, forced expression of Hes-1 causes G_1_-phase cell-cycle arrest. The transcriptional activator E2F-1 is an important cell-cycle regulator, stimulating the G_1_/S-phase transition by activating the transcription of other cell-cycle genes [8]. We earlier identified E2F-1 as a crucial transcription factor directly inhibited by Hes-1 at the transcriptional level in breast cancer [9]. Hes-1 binds to the promoter region of *E2F-1*, thereby repressing its transcription. Based on our findings, we believe that E2F-1 is a central factor in Hes-1-mediated inhibition of proliferation.

Hes-6 is a member of the same family of transcription factors as Hes-1 but functions as a posttranslational inhibitor of Hes-1 [10,11]. Hes-6 forms a heterodimer with Hes-1, thereby preventing its association with transcriptional co-repressors. Hes-6 was first discovered in nervous tissue, but its expression in the mammary gland is not known. Despite its role as an inhibitor of Hes-1, the function of this potential oncogene remains unclear.

Human achaete-scute complex homologue 1 (hASH1) is another member of the bHLH-family. In contrast to Hes-1, hASH-1 functions as a transcriptional activator, inducing transcription through E-boxes, and is negatively regulated by Hes-1 at the promoter level [12,13].

Despite being a potential tumor suppressor *in vitro*, no significant difference in its expression between breast cancer and normal tissue has been found. Therefore, another cofactor is probably involved in the regulation of Hes-1 action.

In an experimental mouse model of colon cancer, several genes were upregulated in metastases, but the only gene that was upregulated in all metastases compared with their primary tumor was Hes-6. Furthermore, the authors showed that Hes-6 is upregulated in several types of human cancers compared with normal tissue [14]. Recently, Hes-6 and hASH-1 have been reported to be overexpressed in high-grade prostate cancer and were suggested to be involved in neuroendocrine development of the cancer cells to an aggressive phenotype [15].

By expressing Hes-6 in the breast cancer cell-line T47D, we studied its role in tumor growth and proliferation. In addition, we investigated its effects on expression of the Hes-1 target gene *E2F-1 *and its potential involvement in ER signaling. Because Hes-6 antagonizes Hes-1, our hypothesis is that Hes-6 increases the proliferation of breast cancer cells and is regulated by estrogen.

## Materials and methods

### Cell cultures

T47D and MCF-7 cells were cultured in DMEM/F12 mixed 1:1, whereas MDA-MB-231 and SK-BR3 cells were cultured in RPMI 1640. Medium was supplemented with 5% fetal bovine serum (FBS). For synchronization of T47D and MCF-7 cells, the medium was changed to phenol red-free DMEM/F12 mixed 1:1 and DMEM, respectively, supplemented with 5% dextran-coated charcoal-treated FBS (DCC) for 24 hours; The serum was then reduced to 0.5% DCC before 10 n*M *ICI 182,780 (ICI) (Tocris, St. Louis, MO, USA) was added.

### Lentivirus vectors and infection of T47D cells

The pLenti6/V5-D-FLAG Hes-6 vector was constructed by cloning a PCR-amplified fragment of Hes-6 into the *Eco*RI site of pcDNA3-FLAG and then amplifying the FLAG-tagged Hes-6 with primers flanked by *Spe*I sites for cloning into the *Spe*I site of pLenti6/V5-D.

Empty pLenti6/V5-D vector, without Hes-6 cDNA, was used as the "mock" control. Lentivirus was produced with the ViraPower Lentivirus Expression system. The titer of the virus was estimated according to instructions (Invitrogen, Carlsbad, CA, USA). T47D cells were seeded onto six-well plates at a density of 200,000 cells per well. The next day, lentivirus at 4 m.o.i was added in 1 ml of growth medium supplemented with 6 μg of hexadimethrine bromide (Polybrene) (Sigma, St. Louis, MO, USA). After 24 hours at 37°C, the cells were washed, and 2 ml of normal growth medium was added. Blasticidine (Invitrogen, Carlsbad, CA, USA) was added to 10 μg/ml after another 24 hours, and the cells were then incubated for another 5 days at 37°C. Non-infected dead cells were washed off, and the remaining cells were trypsinized and expanded for further analysis. All experiments were performed with polyclonal Hes-6-expressing cells.

### Cell-proliferation assay

Then 2,000 cells were plated per well in 96-well plates and cultured in 2% DCC. The next day, cells were synchronized by using 10 n*M *ICI 182780 (Tocris, St. Louis, MO, USA) for 24 hours; the following day, cells were washed once with PBS, and the medium was changed to include the respective treatments (10 n*M *E_2 _or 10 n*M *ICI). After 5 days of incubation, the cell viability was assayed by using the MTS kit (Promega, Madison, WI, USA) (CellTiter 96 Aqueous Non-Radioactive Cell Proliferation Assay). The absorbance was measured at 490 nm.

### Transfection of siRNA to HES-6

The 50,000 cells/well were seeded onto a 24-well plate. The next day, growth medium containing 2% DCC was added. After another 24 hours, the cells were transfected with 50 n*M *siRNA targeting LUC or Hes-6 by using DharmaFECT 2 transfection medium. On the next day, medium containing 0.5% DCC was added, and after another 24 hours, 10 n*M *E_2 _was added to half of the wells. Expression of Hes-6 and E2F-1 was analyzed by using real-time PCR 24 hours after start of treatment.

### Western immunoblotting and antibodies

T47D-Hes-6/mock transduced cells were plated and grown in 150-mm plates until 30% confluence was reached. At time point 0, the cells were incubated with E_2_. Harvesting and extraction were done at 24 hours according to standard protocol. SDS-PAGE was performed as described [16]. The following primary antibodies were used: E2F-1 (sc-251, Santa Cruz Biotechnology, Santa Cruz, CA, USA), β-actin (Sigma, St. Louis, MO, USA), and Hes-6 (rabbit antibody raised in our laboratory).

### RNA extraction and real-time PCR

RNA extraction was performed with TRIzol reagent (Invitrogen, Carlsbad, CA, USA) and chloroform extraction, according to standard protocol. cDNA synthesis was performed with the First Strand System according to standard protocol (Nordic Bioservice AB, Stockholm, Sweden). Real-time PCR was performed with NBS Mastermix (Nordic Bioservice AB, Stockholm, Sweden). The following primers and probes were used: 18s rRNA F: 5'-CCT GCG GCT TAA TTT GAC TCA-3', R: 5'-AGC TAT CAA TCT GTC AAT CCT GTC C-3' as a reference gene. The real-time PCR reactions were performed in an ABI PRISM 7500 (Applied Biosystems, Carlsbad, CA, USA) with optimized conditions for NBS Mastermix: 50°C for 2 minutes, 95°C for 10 minutes, followed by 40 to 50 cycles at 95°C for 15 seconds and 60°C for 50 seconds. The optimal concentration of primers was determined in preliminary experiments, and all SYBR-Green primer pairs were checked with dissociation-curve analysis.

### Experimental animals and xenograft model

Mice were housed in filter-top cages under sterile conditions at the animal facility at Karolinska University Hospital, Huddinge. One confluent 150-cm^2 ^Falcon cell-culture flask per mouse with T47D-Hes-6 or T47D-mock cells was trypsinized and diluted with 200 μl normal medium + 200 μl Matrigel (BD Falcon, San Jose, CA, USA). The cell suspension (300 μl) was injected into the abdominal fat close to the mammary tissues of 9- to 12-week-old pathogen-free SCID/beige mice (Taconic, Lillie Skensved, Denmark) on day 0. E_2 _pellets, 0.72 mg/pellet (IRA, Sarasota, FL, USA), were injected subcutaneously in the neck with a pellet trochar (IRA, Sarasota, FL, USA). After 7 and 14 days, the mice were killed, and the tumor volume was measured with calipers according to the formula length × width × height. All tumors were fixed in 4% paraformaldehyde and stored in 75% ethanol, and tumors of sufficient size also were prepared for mRNA extraction as described later. Animal experiments were approved by Swedish Board of Agriculture, reference number: S 27-08, including approved animal welfare, experimental protocol, and animal toxicology.

### Human breast cancer samples

Human breast samples were obtained from breast cancer surgery performed at the Hammersmith Hospital, London, UK, between the years 1985 and 1998. All tissues were made anonymous for the researchers, and identities could not be connected to individual samples. To each tissue sample, the following parameters were attached: Tumor size, tumor stage, age at diagnosis, age of patient, and menopausal status. The samples consisted of 28 normal or benign breast tissues (control) and 38 breast cancer tissues. All tissue samples were homogenized, RNA-extracted, and subsequently analyzed with real-time PCR according to procedure. The study was ethically approved by Forskningsetiska nämnden, Stockholm, reference number 04-951/1. All tumor samples were anonymous and cannot be tracked to the corresponding patient; therefore, an informed consent from each patient was deemed unnecessary by the ethics committee.

### Ki67-immunohistochemistry

To examine the number of proliferating cells in tumor xenografts, immunohistochemistry with a Ki-67 antibody was performed. Antigen retrieval was done by microwave boiling in 0.01 *M *citric acid (pH 6.0) for 120 seconds, and then slides were left undisturbed for 20 minutes. Endogenous peroxidase activity was blocked by incubating sections shaking in 3% hydrogen peroxide in methanol for 10 minutes and then blocked in 2% bovine serum albumin (BSA) and 0.1% NP40 for 1 hour at room temperature. Primary antibody Ki-67 (DakoCytomation, Glostrup, Denmark) was diluted 1:100 in 2% BSA and 0.1% NP40 and incubated overnight at 4°C. Sections were then washed consecutively in PBS, 0.1% NP40 for 3 × 5 minutes, followed by incubation with a biotinylated antibody BA-9200 1:200 in 0.1% NP40 for 1 h (Vector Laboratories, Burlingame, CA, USA). After this, sections were incubated in the streptavidin-horseradish peroxidase ABC complex (Vectastain Elite; Vector Laboratories, Burlingame, CA, USA) for 1 hour, stained in DAB, and counterstained with Mayer hematoxylin (Sigma, St. Louis, MO, USA) before dehydration through ethanol, and mounted in Pertex (Histolab, Gothenburg, Sweden). The number of Ki-67-positive cells was counted on two independent fields per slide with a 20× objective. The average number of positive cells and SD were calculated for each group.

### Statistics

Values are expressed as means with 95% confidence intervals. An unpaired, two-tailed *t *test was used to compare differences between two groups. One-way ANOVA with Tukey's multiple-comparisons posttest was used to analyze differences between three or more groups. Significance is presented as *P < 0.05, **P < 0.005, ***P < 0.001; nonsignificant differences are presented as NS.

## Results

**Figure 1:**
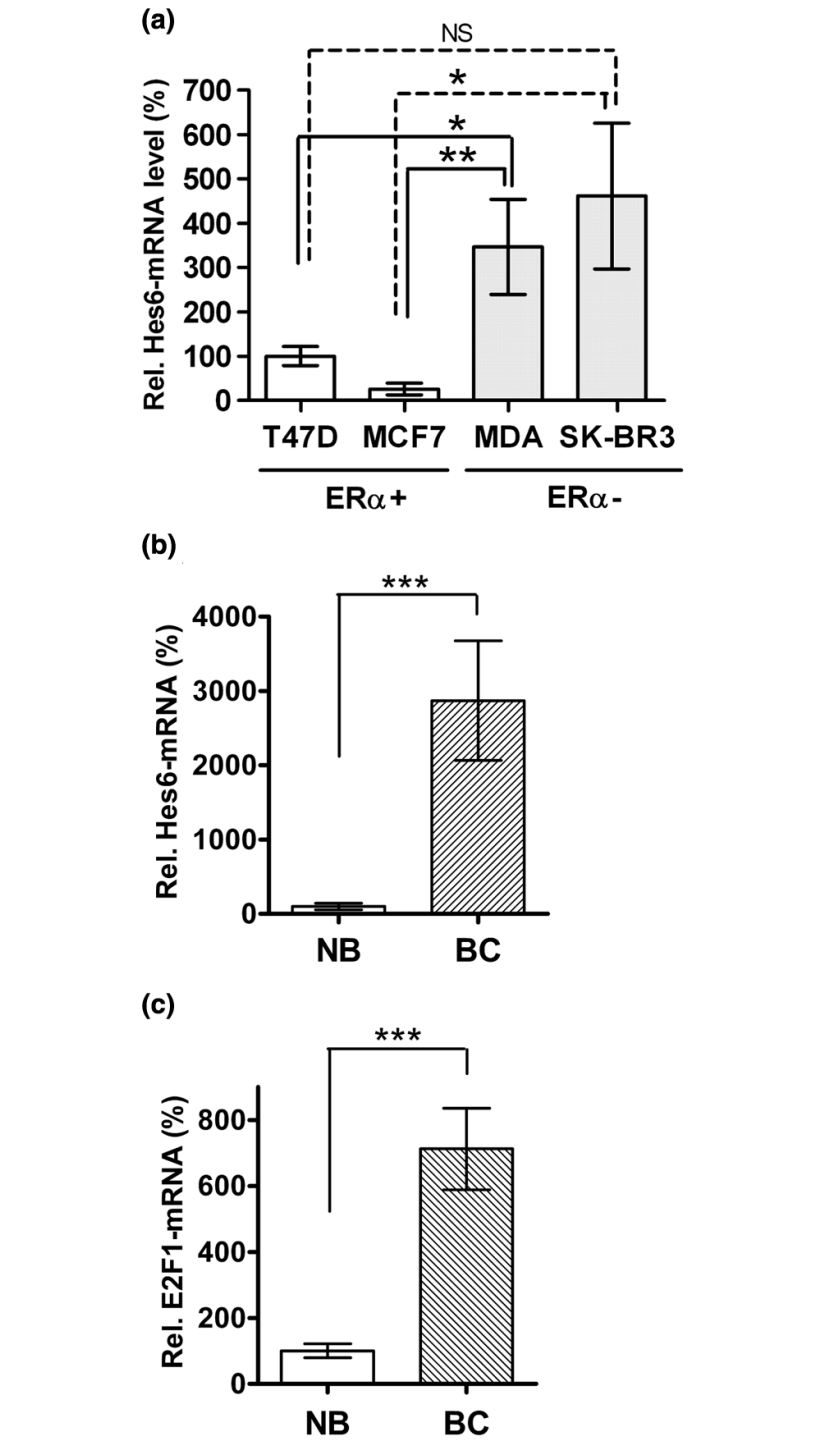
Expression of Hes-6 is increased in breast cancer. **(a) **Expression of Hes-6 mRNA in breast cancer cell lines T47D (bar 1), MCF-7 (bar 2), SKBR3 (bar 3), and MDA-MB-231 (bar 4). All experiments were repeated twice with similar results. Data are presented as mean values of six samples (bars 1, 2, and 3) and five samples (bar 4) ± SEM. Expression of Hes-6 mRNA **(b) **and E2F-1 mRNA **(c) **in noncancerous breast biopsies (bar 1) compared with malignant breast (bar 2). Data are presented as mean values of 24 (bars 1) and 28 (bars 2) samples ± SEM. Graphs are shown as relative values to bars 1 (100%) of each graph. Significance is presented as *P < 0.05; **P < 0.005; ***P < 0.001. Nonsignificant differences are presented as NS.

Elevated levels of Hes-6 have been found in prostate and colon cancers compared with normal tissue, where the expression is low. We first performed real-time PCR to determine Hes-6 mRNA levels in different breast cancer cell lines. The estrogen-dependent, ERα-positive T47D and MCF-7 cells contained low levels of Hes-6, in comparison with the more-aggressive, ERα-negative breast cancer cell lines MDA-MB-231 and SKBR3, which contained 4 to 10 times higher levels of Hes-6 mRNA (Figure 1a).

We investigated the expression of Hes-6 in normal breast tissue compared with cancer tissue. The expression of Hes-6 mRNA was very low in normal breast specimens. In breast cancer tissue, however, Hes-6 expression was increased by more than 28 times compared with that in normal breast specimens (Figure 1b). The cell-cycle regulator E2F-1 was also significantly increased in breast cancer samples compared with normal breast tissue (Figure 1c).

**Figure 2:**
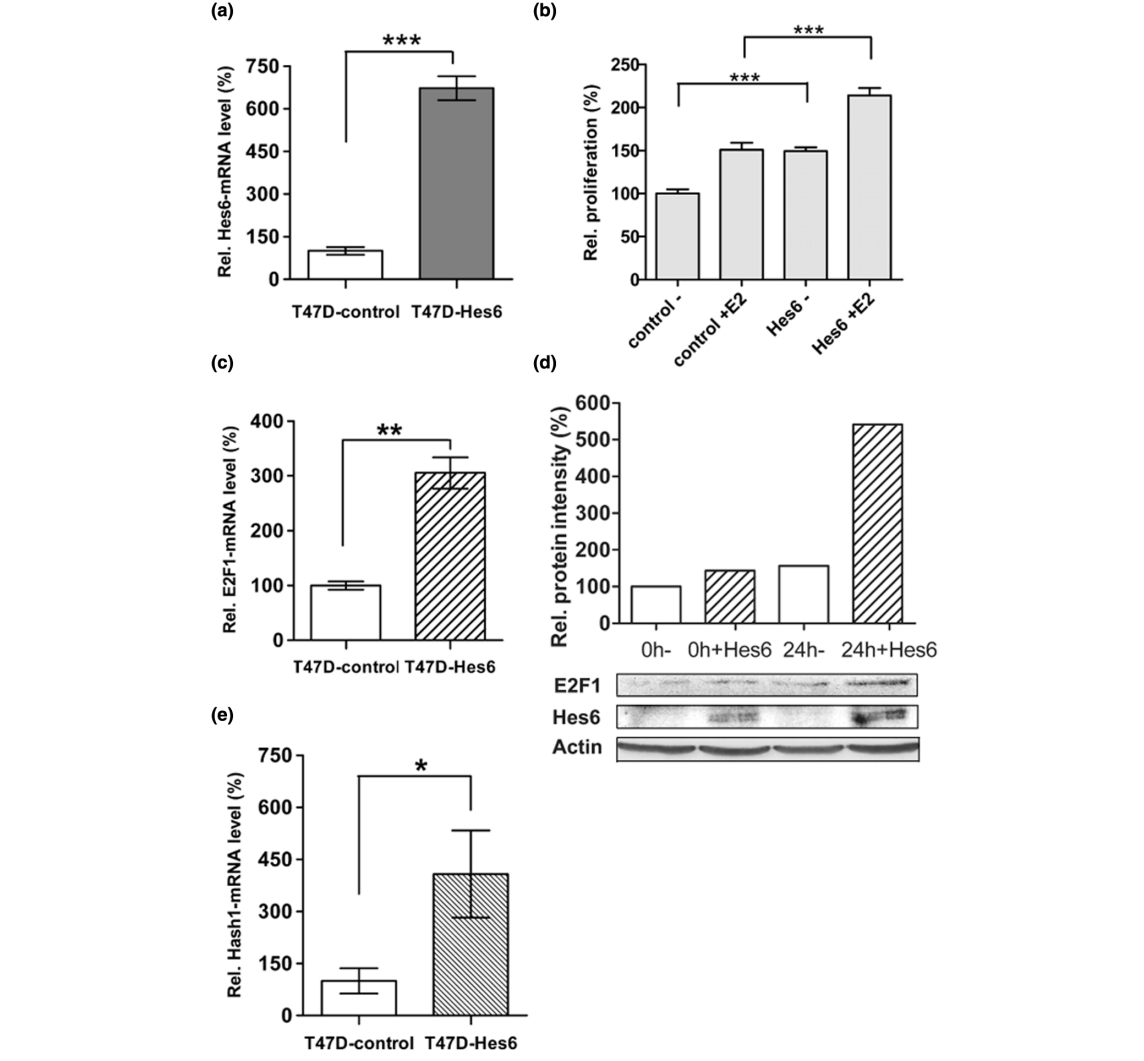
Hes-6 increases proliferation and E2F-1 transcription in T47D cells. **(a) **Expression of Hes-6 mRNA in T47D-control (bar 1) and T47D-Hes-6 cells (bar 2) cells (three samples/bar). **(b) **Proliferation assay performed with MTS kit (Promega) after 5 days' incubation ± E_2 _on synchronized, polyclonal T47D-control/Hes-6 cells. T47D-control cells without (bar 1) or with (bar 2) E_2_, T47D-Hes-6 cells without (bar 3) or with (bar 4) E_2 _(12 samples/bar). **(c) **Relative E2F-1 mRNA levels in T47D-control (bar 1) and T47D-Hes-6 (bar 2) cells (three samples/bar) in nonsynchronized cells. **(d) **E2F-1 (upper lane) and Hes-6 protein (lower lane) in synchronized T47D-control/Hes-6 cells, measured with Western blot. Graphs represent protein intensity of E2F-1 Western blot, analyzed with Biorad software; T47D-control (bar 1) and T47D-Hes-6 at 0 hours (bar 2) in the absence of E_2_, T47D-control (bar 3), and T47D-Hes-6 at 24 hours (bar 4) in the presence of E_2_. **(e) **Relative hASH-1 mRNA levels in T47D-control (bar 1) and T47D-Hes-6 (bar 2) cells (three samples/bar) in nonsynchronized cells. Graphs are shown as relative values to bars 1 (100%) of each graph. All graphs are presented as mean values ± SD. Significance is presented as *P < 0.05; **P < 0.005; and ***P < 0.001.

Because Hes-6 is expressed at low levels in ERα+ breast cancer cells, we generated stable Hes-6-expressing T47D cells by lentiviral transduction with a Hes-6 expression vector (Figure 2a).

We showed previously that Hes-1 represses the proliferation of breast cancer cells and that E2F-1 is directly inhibited by Hes-1 at the transcriptional level. The transcription factor E2F-1 is a central player in the regulation of proliferation and functions by activating transcription of G_1_/S/G_2_-phase genes. Because Hes-6 is an inhibitor of Hes-1, we reasoned that Hes-6 would affect proliferation as well as E2F-1 expression.

T47D cells expressing Hes-6 or with empty vector as control (T47D-Hes-6/control) were synchronized and incubated without E_2 _for 24 hours. After 5 days, proliferation assays were performed by incubating the cells with the tetrazolium compound MTS, and subsequently analyzed by measuring the absorbance of the cell-culture medium at 490 nm (MTS assay).

As shown in Figure 2b, E_2 _treatment of T47D control cells increased proliferation by approximately 50%. Expression of Hes-6 in the absence of E_2 _also increased proliferation to about the same extent. E_2 _treatment of T47D-Hes-6 cells caused a further stimulation of proliferation by approximately 30%.

Real-time PCR and Western blot were performed on T47D-Hes-6 or mock cells to investigate the transcriptional effects of Hes-6. We found that the expression of Hes-6 resulted in induction of E2F-1 mRNA and protein levels in comparison to the control (Figure 2c and 2d). The Hes-1 target hASH-1, which is normally inhibited by Hes-1, was strongly upregulated at the mRNA level in response to Hes-6 expression (Figure 2e).

**Figure 3:**
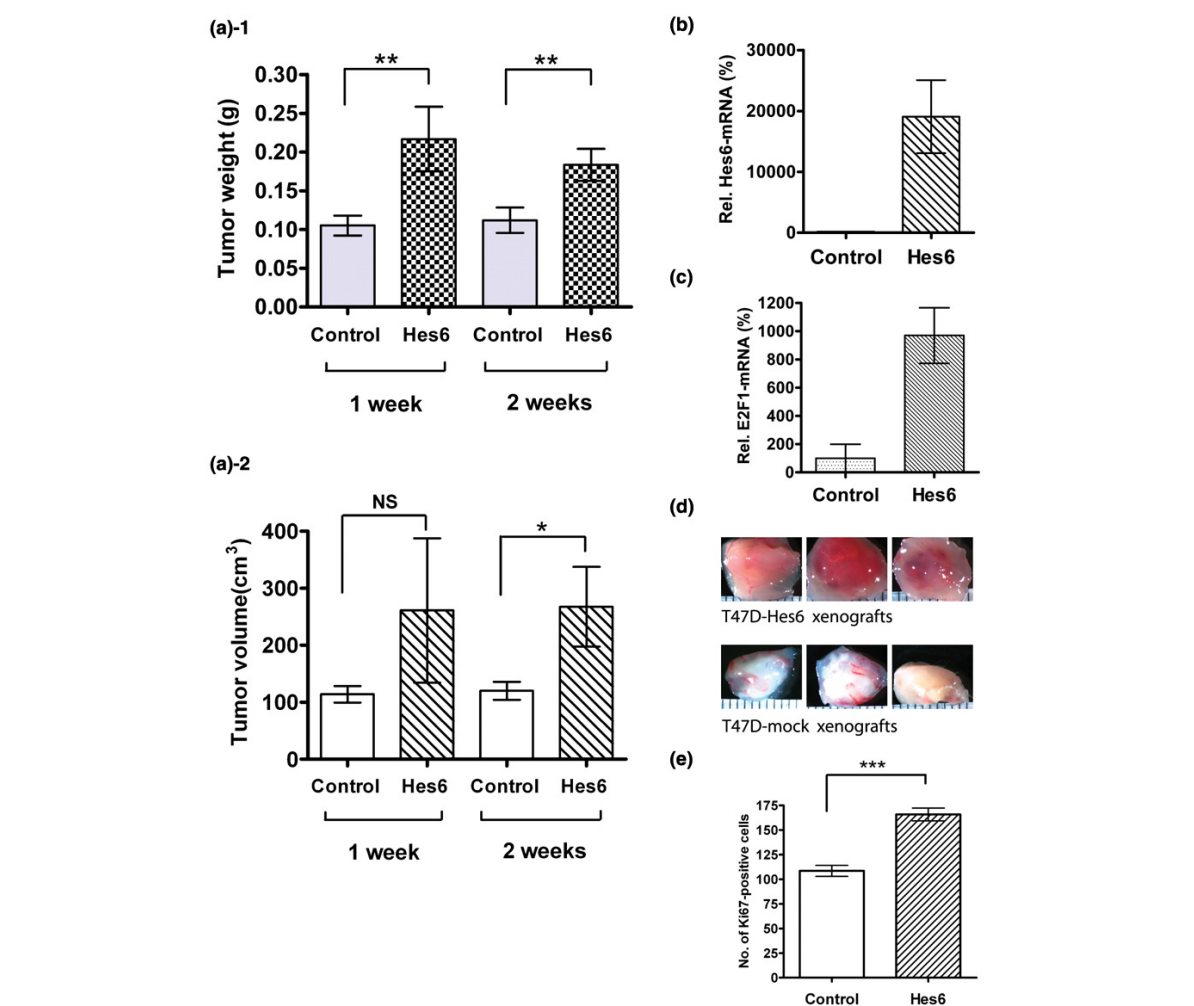
Hes-6 expression increases the growth of T47D xenografts. **(a) **Tumor weight (**a-1**) and volume (**a-2**) of T47D-control (bars 1, 3) (n = 4 and 5, respectively) and T47D-Hes-6 xenografts (bars 2 and 4) (n = 3 and 4, respectively) in SCID/beige mice measured after 7 (bars 1, 2) and 14 days (bars 3, 4). Data are presented as mean ± SD. Relative Hes-6 **(b) **and E2F-1 mRNA-levels **(c) **in T47D-mock (bar 1) xenografts (set as 100%) and T47D-Hes-6 xenografts (bar 2). Data are presented as mean values of eight (bar 1) and six (bar 2) samples ± SD: **(d) **Representative pictures of T47D-Hes-6 xenografts (upper lane) or T47D-mock xenografts (lower lone) grown for 1 week. **(e) **Number of Ki67-positive cells per visual field (20× objective) in control xenografts (bar 1) and Hes-6-expressing xenografts (bar 2). Data are presented as means of eight xenografts per bar. Significance is presented as *P < 0.05; **P < 0.005; and ***P < 0.001. Nonsignificant differences are presented as NS.

Further to study the function of Hes-6 in breast cancer cells and to determine whether Hes-6 influences tumor growth, we performed xenograft studies in SCID/beige-immunodeficient mice. The T47D-Hes-6 or control cells were injected into the abdominal subcutaneous fat of 12-week-old female mice. Mice were killed on day 7 or 14, and the tumors were taken out, measured, and weighed (Figure 3a); representative pictures are shown in Figure 3d. Expression of Hes-6 caused a significant increase in both tumor weight (Figure 3a-1) and size (Figure 3a-2) at both 7 and 14 days. To measure the rate of proliferation *in vivo*, Ki67-immunohistochemistry was performed on the xenograft sections. Although the proliferation was high in both groups, the number of Ki67-positive cells was significantly higher in Hes-6yexpressing xenografts compared with controls (Figure 3e). RNA was extracted from the tumors and analyzed with real-time PCR. Whereas the endogenous level of Hes-6 in the T47D-control tumors was low, a strong induction of Hes6 was found in the T47D-Hes-6 tumors (Figure 3b). In addition, E2F-1 was upregulated at the mRNA level in the Hes-6-expressing tumors compared with the controls (Figure 3c), in line with the *in vitro *experiments. Notably, however, the induction of E2F-1 was stronger in the xenografts compared with the *in vitro *cultured cells.

**Figure 4:**
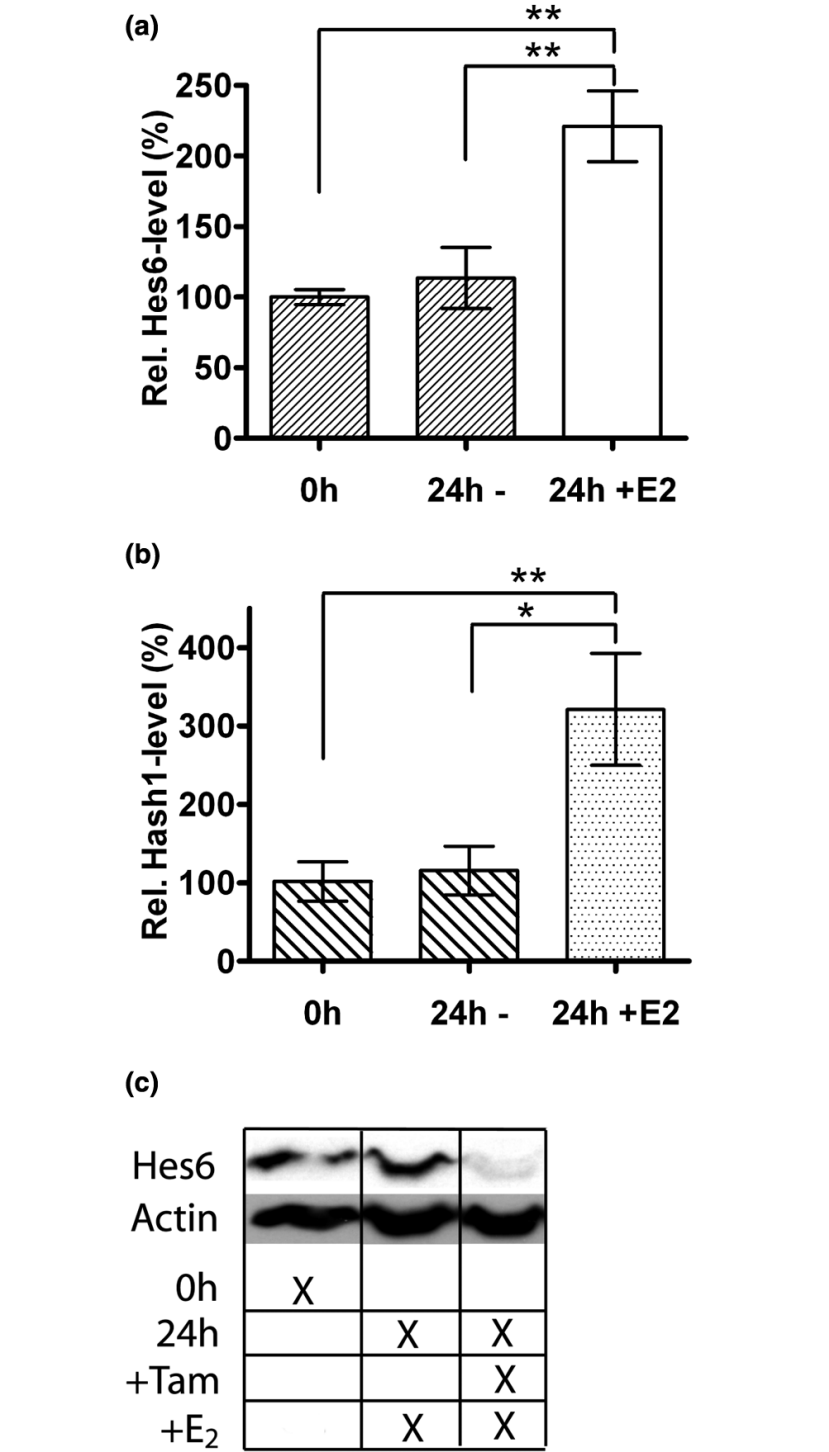
Hes-6 is expressed in response to ERα stimulation in MCF-7 cells. **(a) **Relative Hes-6 mRNA levels in synchronized, non-E_2_-treated MCF-7 cells (bar 1), non-E_2_-treated MCF-7 cells after 24 hours (bar 2), and E_2_-treated MCF-7 cells after 24 hours (bar 3). **(b) **Relative hASH-1 mRNA levels in synchronized, non-E_2_-treated MCF-7 cells (bar 1), non-E_2_-treated MCF-7 cells after 24 hours (bar 2), and E_2_-treated MCF-7 cells after 24 hours (bar 3). All data are presented as mean values ± SD of three samples/bar. **(c) **Hes-6 protein in synchronized MCF-7 cells without E_2_ (column 1), treated with E_2 _(column 2), and with both E_2 _and tamoxifen (column 3) for 24 hours.

Estrogen-bound ERα is an important stimulator of proliferation in breast cancer cells [5] and is consequently an important target for endocrine therapy of breast cancer. ERα drives proliferation by stimulating several cell-cycle factors [17], but also by inhibiting the expression of Hes-1, as described earlier [7]. We wanted to see whether ERα also could influence the expression of Hes-6. Through measuring Hes-6 mRNA and protein in E_2_-treated synchronized MCF-7 cells, we observed that Hes-6 mRNA was upregulated in response to E_2 _treatment (Figure 4a), indicating that ERα activates Hes-6 expression in the presence of E_2_. E_2 _treatment also caused a robust induction of hASH-1 mRNA (Figure 4b). At the protein level, Hes-6 was induced in response to E_2 _(Figure 4c). Consistently, treatment with tamoxifen reduced the Hes-6 level dramatically.

**Figure 5:**
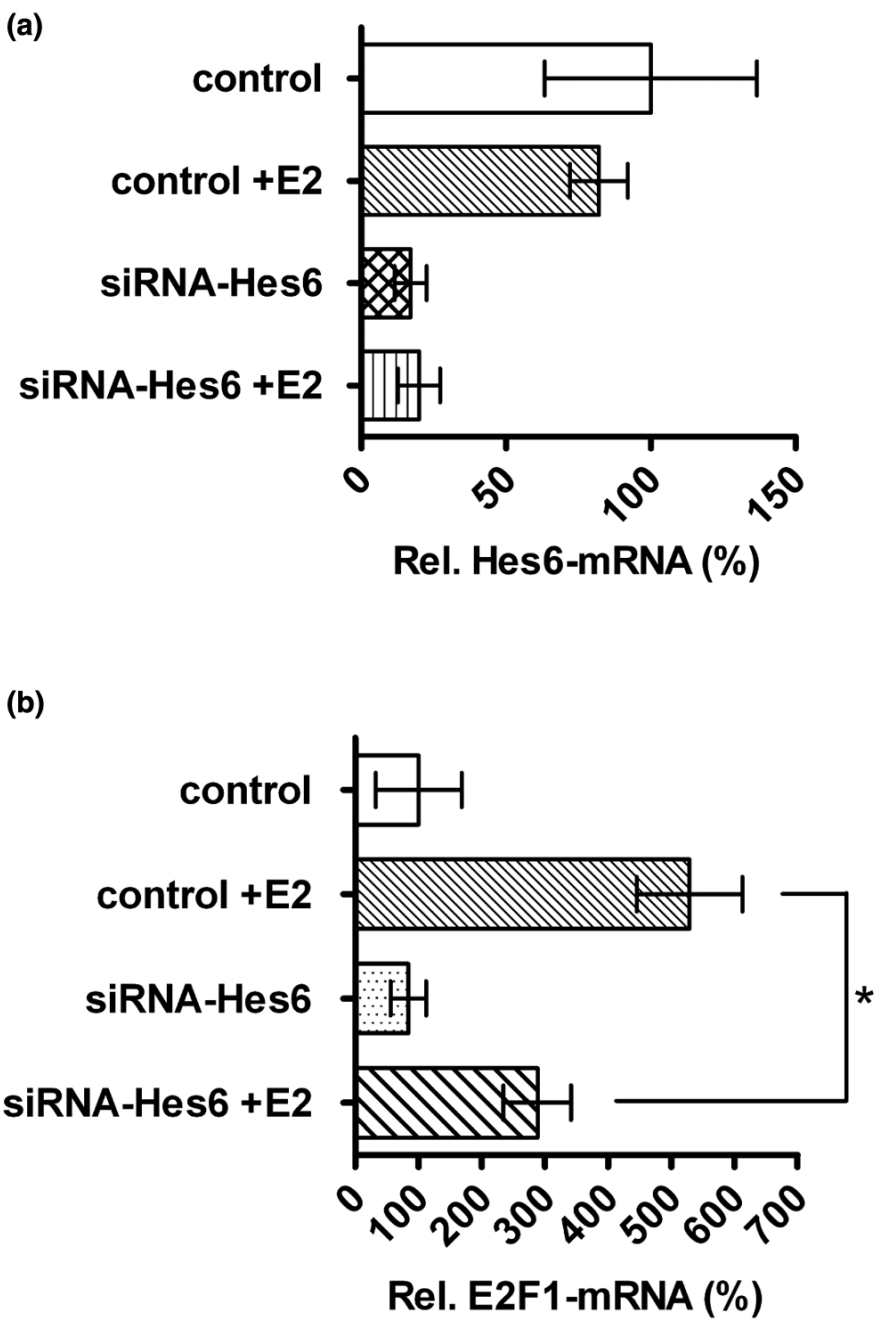
siRNA against Hes-6 causes a reduction of E2F-1 mRNA. Relative Hes-6 **(a) **and E2F-1 mRNA levels **(b) **in MCF-7 cells transfected with 50 n*M *siRNA against Hes-6 (siRNA-Hes-6) or luciferase as control (control). Graphs are presented as values relative to MCF-7 cells transfected with the siRNA luciferase control vector. All bars represent mean values ± SD of three samples, and experiments were repeated twice with similar results. Significance is presented as *P < 0.05.

Finally, we used siRNA against Hes-6 to study whether E2F-1 expression is dependent on Hes-6 expression. Transfection of MCF-7 cells with 50 n*M *Hes-6 siRNA reduced Hes-6 mRNA level in both presence and absence of E_2 _(Figure 5a). However, siRNA against Hes-6 did not reduce the E2F-1 mRNA level in absence of E_2_, but counteracted the E_2_-mediated upregulation of E2F-1 (Figure 5b), compared with the siRNA-luciferase control.

## Discussion

We previously identified and characterized Hes-1 as an essential transcription factor in ERα-signaling and a strong inhibitor of estrogen-stimulated proliferation in breast cancer cells [7]. However, despite several attempts to investigate Hes-1 expression in breast cancer, we have not found any difference in Hes-1 expression in breast cancer tissues compared with normal tissues. For this reason, we investigated the expression of the Hes-1 inhibitor Hes-6 and its potential involvement in proliferation.

As described in the Introduction, the transcription factor Hes-6 is a novel Hes-family member, whose only established function is to inhibit DNA binding of Hes-1, thereby inhibiting Hes-1 activity. Here we identify *Hes-6 *as a novel estrogen-regulated gene in breast cancer. Most interestingly, as shown in Figure 1, Hes-6 is expressed at higher levels in breast cancer tissue compared with normal breast tissue. This result could be interpreted in at least two different ways: Hes-6 could be a marker associated with breast cancer in general, without any major function. Alternatively, it could mean that Hes-6 is induced in early stages of breast cancer and is important for the progression of breast cancer. In this study, we present evidence that Hes-6 has an important role in the proliferation of breast cancer cells and in the growth of corresponding xenografts.

As expected, in parallel to the high levels of Hes-6, we identified a higher expression of E2F-1 in the breast cancer samples compared with that in the normal breast tissue samples (Figure 1c), in agreement with what has been described by other researchers. Based on our findings, we suggest that increased E2F-1 levels could be a result of regulation by the Hes-1/Hes-6 system.

As shown in Figure 1a, Hes-6 mRNA also was higher in the aggressive, ERα-negative cell lines SKBR3 and MDA-MB231 than in the ERα-positive T47D and MCF-7 cells. Because Hes-6 has been identified as a marker for aggressive, high-grade cancers of other origins than the breast, Hes-6 also could be associated with high proliferation and aggressiveness in breast cancer. Conversely, this finding is in contrast to our experiments implicating *Hes-6 *as a gene expressed in response to ERα stimulation (Figure 4). It is likely that other, ER-independent signaling pathways also regulate Hes-6 expression. This is exemplified by the nerve growth factor activity of Hes-1 during differentiation of the pheochromocytoma cell line PC12 [18]. Because genetic abnormalities are common in breast cancer [19], it also is possible that the DNA regions harboring the *Hes-6 *gene are amplified in some breast cancer cells, thereby causing constantly increased Hes-6 levels.

The function of Hes-6 has been studied extensively within the developing nervous system [10,11]. Moreover, the expression of Hes-6 in different tissues has been reported. Nevertheless, this is the first study in which the function of Hes-6 in cancer is described.

As shown in Figure 3, expression of Hes-6 caused increased proliferation in both the absence and the presence of E_2_, The most probable explanation is that Hes-6 inhibits Hes-1, thereby changing the expression of cell-cycle regulators. E2F-1 might be particularly important in this context, because it is strongly inhibited by Hes-1 at the transcriptional level [9]. In agreement with this notion, expression of Hes-6 resulted in induction of E2F-1 at both the mRNA and the protein levels, whereas inhibition of Hes-6 by siRNA prevented the ERα-mediated induction of E2F-1 (Figures 2 and 5).

In the xenograft experiments shown in Figure 3, the expression of Hes-6 resulted in a dramatic increase of tumor growth, most likely as a consequence of the proliferation-stimulatory function of Hes-6. However, expression of Hes-6 might also affect xenograft growth in proliferation-independent pathways; for example, Hes-6 might regulate angiogenesis and paracrine growth factors. When Hes-6 was expressed in T47D cells, the levels of both Hes-6 and E2F-1 was higher in the cells grown as xenografts than in *in vitro *cultured cells (Figures 2 and 3). A possible reason for this is that the three-dimensional *in vivo *milieu and the presence of stromal factors stimulate Hes-6 expression further. We believe that alternative pathways can at least partially overcome ER regulation in subtypes of breast cancer.

The important role of E2F-factors in cancer has been investigated by other researchers in great detail [8,20]. E2F-1 has been studied as a potential marker in breast cancer diagnostics. In one report, E2F-1 protein correlated with Mib1/Ki67 expression and was expressed at higher levels in advanced-stage breast cancer [21]. High expression of E2F-1 also has been shown in small cell lung cancer, and these tumors do not express Hes-1. Conversely, non-small cell lung cancer expresses lower levels of E2F-1 and higher levels of Hes-1, indicating that Hes factors may be important in E2F-1 regulation in these cancers as well [22].

As shown in Figure 4, E_2 _treatment of MCF-7 cells caused increased expression of Hes-6 mRNA and protein. Because MCF-7 cells contain only ERα, this effect should be mediated through ERα. However, transient transfections of T47D and MCF-7 cells with a Hes-6 promoter construct did not reveal any ERα-mediated regulation (data not shown). Accordingly, we speculate that an element upstream or downstream of the proximal *Hes-6 *promoter is responsible for ERα-regulated Hes-6 transcription.

In addition to Hes-6, we found that E_2 _treatment of MCF-7 cells resulted in increased hASH-1 expression. Moreover, hASH-1 was expressed in response to Hes-6 in T47D cells.

Because *hASH-1 *is normally repressed by Hes-1 through an element in its proximal promoter [23], it is likely that the induction of *hASH-1 *is a consequence of Hes-6 expression, leading to inactivation of Hes-1. In tissues, Hes-6 and hASH-1 expression are often associated with each other. For instance, it was recently shown that Hes-6 and hASH-1 correlate with more-aggressive prostate cancer [15].

As shown in an earlier study, Hes-1 is repressed by ERα at the transcriptional level [16]. We therefore suggest that E_2 _treatment of ERα^+ ^breast cancer cells leads to inactivation of Hes-1, both directly and through the induction of Hes-6. However, the role of ERβ in Hes-6 regulation is not known and must be clarified in future studies. Interestingly, as shown in Figure 4, treatment of MCF-7 cells with the selective estrogen-receptor modulator (SERM) tamoxifen caused repression of Hes-6, indicating that Hes-6 might work as a marker for tamoxifen response in breast cancer cells. In addition, it is possible that repression of Hes-6 could be important for the breast cancer-suppressive effects of tamoxifen.

## Conclusions

Based on our findings, we propose that Hes-6 has an important role in the proliferation of breast cancer cells. Hes-6 is expressed in low levels in normal breast tissue but is strongly induced in breast cancer tissue. As an ERα-regulated gene, Hes-6 constitutes a novel link between estrogen signaling and the Hes family of proteins, which are involved in differentiation and proliferation. Consequently, a better knowledge of this signaling pathway could be important for the identification of endocrine-resistant tumors. Furthermore, Hes-6 seems to be essential in ERα-mediated induction of E2F-1, a critical step in the G_1_/S-phase transition of the cell cycle. Hes-6 emerges as a potential marker for breast cancer and might be a target for novel treatments based on the Hes signaling pathway.

## Abbreviations

bHLH: basic helix-loop-helix; E_2_: 17β-estradiol; ER: estrogen receptor; hASH-1: human achaete-scute complex homologue 1; Hes: hairy and enhancer of split; SCID: severe immunodeficiency.

## Competing interests

The authors declare that they have no competing interests.

## Authors' contributions

JH and AS carried out experimental studies. EWFL provided clinical breast tumor samples. JH, AS, and JÅG designed the study. JH, AS, EWFL, and JÅG prepared the manuscript. All authors read and approved the final manuscript.
